# Sustainable Electrically Conductive Bio-Based Composites via Radical-Induced Cationic Frontal Photopolymerization

**DOI:** 10.3390/polym16152159

**Published:** 2024-07-30

**Authors:** Dumitru Moraru, Alejandro Cortés, David Martinez-Diaz, Silvia G. Prolongo, Alberto Jiménez-Suárez, Marco Sangermano

**Affiliations:** 1 Dipartimento di Scienza Applicata e Tecnologia, Politecnico di Torino, C.so Duca degli Abruzzi 24, 10129 Torino, Italy; dumitru.moraru@polito.it; 2 Materials Science and Engineering Area, University Rey Juan Carlos, C/Tulipán s/n, 28933 Madrid, Spain; alejandro.cortes@urjc.es (A.C.); david.martinez.diaz@urjc.es (D.M.-D.); silvia.gonzalez@urjc.es (S.G.P.); alberto.jimenez.suarez@urjc.es (A.J.-S.); 3 Instituto de Tecnologías para la Sostenibilidad, University Rey Juan Carlos, C/Tulipán s/n, 28933 Madrid, Spain

**Keywords:** diglycidylether of vanillyl alcohol (DGEVA), radical-induced cationic frontal photopolymerization (RICFP), recycled carbon fibers (RCFs), conductive, joule effect

## Abstract

Diglycidylether of vanillyl alcohol (DGEVA), in combination with mechanically recycled carbon fibers (RCFs), was used to make, via Radical-Induced Cationic Frontal Photopolymerization (RICFP), fully sustainable and bio-based conductive composites with good electrical conductivity and consequent Joule effect proprieties. Three different fiber lengths, using three different sieve sizes during the mechanical recycling process (0.2, 0.5, and 2.0 mm), were used in five different amounts (ranging from 1 to 25 phr). The samples were first characterized by dynamic mechanical thermal analysis (DMTA), followed byelectrical conductivity and Joule heating tests. More specifically, the mechanical properties of the composites increased when increasing fiber content. Furthermore, the composites obtained with the longest fibers showed the highest electrical conductivity, reaching a maximum of 11 S/m, due to their higher aspect ratio. In this context, the temperature reached by Joule effect was directly related to the electrical conductivity, and was able to reach an average and maximum temperatures of 80 °C and 120 °C, respectively, just by applying 6 V.

## 1. Introduction

In the pursuit of more sustainable and environmentally friendly producing methods for polymeric materials, it is imperative that not only the production process be energy-efficient and low in emissions, but also that the raw materials used are sustainable. There is a big effort in the research towards the development of bio-based polymeric matrices, which are usually derived from biomass [1,2,3,4,5,6,7,8,9,10].

In fact, environmental concerns have been rising for several decades now, and the urgency of climate change is more pressing than ever. Within this context, it is necessary to limit the use of fossil-based resources. The exploitation of the biomass from plants can represent an attractive source of polymeric bio-based precursors [3,11,12,13]. A huge amount of work is already reported in the literature, where bio-based precursors are exploited to produce both linear polymers, such as polyester, polyurethane and polycarbonates, as well as crosslinked resins, such as vinyl ester, epoxy, and acrylate resins [4,14,15,16,17], which are very important in the production of composites.

On the other hand, the alternative pathway for sustainability is the recycling and valorization of industrial waste. When focusing on polymeric composites, the use of recycled fibers as reinforcing agents could be interesting when couple with the use of a bio-based matrix achieved from derivatization of agro-food waste products.

The recovery and recycling of carbon fiber has witnessed a significant attention in recent years, both because the fibers can be recycled at a very low costs [18], and due to the large volume of manufacturing waste and upcoming end-of-life products that will enter the waste stream [19,20,21]. The mechanically recycled carbon fibers retain most of the original mechanical properties, but their length is generally reduced [22]. The recycling of carbon fibers putting them back into the track of circular economy.

Currently, most thermoset composites are produced through thermal polymerization, which involves the use of large ovens and autoclaves which, consequently, involves high energy consumption. Given this premise, several strategies can be employed to mitigate the environmental impact of the production process. UV curing is a promising technique for thermosets production due to its high efficiency in energy consumption and low VOC emissions [23,24]. However, it does have its limitations, particularly when light penetration into the polymer matrix is restricted, especially when the matrix is reinforced with opaque fillers, such as carbon fibers. To overcome those drawbacks, the UV-induced frontal polymerization (FP) technique can be exploited.

**Scheme 1:**
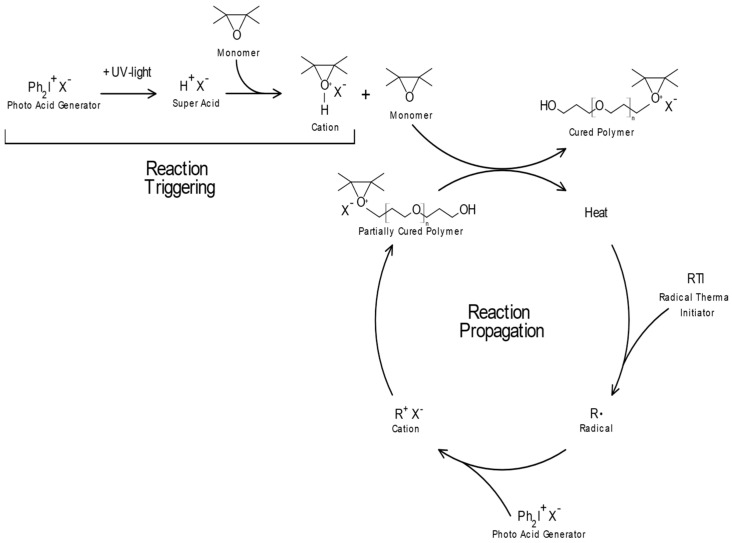
Reaction scheme of the Radical-Induced Cationic Frontal Polymerization.

The photo-induced FP process can be either radical or cationic. Since most polymeric matrices exploited for composites are epoxy-based resins, the cationic UV-induced frontal polymerization process has been deeply investigated [25,26,27,28,29,30]. The process involves the photogeneration of a superacid, starting from an iodonium salt, which enables the surface, ring-opening polymerization process. The heat release during cationic ring-opening polymerization will cleave the thermo-labile initiator generating reactive radicals, which are oxidized in the presence of the iodonium salt, forming a reactive carbocation [26,27,31,32,33]. The entire process is a cycle, and curing reactions proceed towards the thickness of the samples, since the heat front will be sustained (see Scheme 1). Because of the thermal-frontal radical formation, which is subsequently oxidized to carbocation, the process was named Radical-Induced Cationic Frontal Polymerization (RICFP).

In this study, the RICFP technique was used to cure a bio-based epoxy resin reinforced with mechanically recycled carbon fibers (RCFs), with the aim of achieving electrically conductive fully bio-based composites that show Joule effect capability.

## 2. Materials and Methods

**Figure 1:**
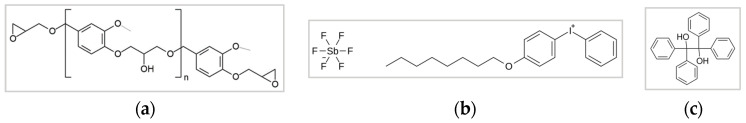
DGEVA structure (**a**), PIFA—cationic photoinitiator structure (**b**), TIPED structure (**c**).

*Materials*: The bio-based epoxy monomer, Diglycidylether of vanillyl alcohol (DGEVA), was supplied by Specific Polymers. The thermal initiator, 1,1,2,2-tetraphenyl-1,2-ethanediol (TPED), was procured from Sigma Aldrich. The cationic photoinitiator, (p-octyloxyphenyl)phenyliodonium hexafluoroantimonate (PIFA), was acquired from ABCR. All of the chemical structures are reported in Figure 1. The recycled carbon fibers (RCFs) were obtained from expired offcuts generated during the manufacturing process of continuous fiber-reinforced polymers (CFRP) from carbon fiber-epoxy prepregs. All materials and chemicals were utilized as received.

*Preparation of RCFs*: The expired prepreg off-cuts were introduced into a cutting mill (Fritsch Pulverisette 19, Fritsch, Idar-Oberstein, Germany) to reduce the carbon fiber (Hexcel IM5-12K, Milwaukee, WI, USA) length. This step is repeated five times at 3000 r.p.m. to achieve a homogeneous recycled product. The prepregs were cleaned in acetone for 5 min before the milling process, aiming to reduce the remaining partially cured epoxy resin content. Additionally, three different sieves, with pore sizes of 0.2, 0.5, and 2.0 mm, respectively, were placed inside the mill to obtain RCFs with different characteristics.

*Preparation of bio-based composite*: Thermoset composites were fabricated using recycled carbon fibers (RCFs). Pure DGEVA was combined with 1 phr (parts per hundred resin) of TPED, and a [TPED]/[PIFA] = 1.0 mol/mol was maintained [25]. Subsequently, varying quantities of RCFs, ranging from 1 to 25 phr, were incorporated. To facilitate the dissolution of the photoinitiator and the dispersion of the fibers, the formulation was sonicated for 10 min at 60 °C. Samples of 10 × 10 × 1 mm were prepared in silicon molds. The mold’s surface was exposed to UV light to promote the UV-induced frontal polymerization process. A Hamamatsu LC8 lamp was used, which has a light intensity of 100 mW/cm^−2^.The UV lamp’s emission spectrum ranged from 275 to 500 nm, with a peak at 365 nm.

Refer to Table 1 for the prepared and tested formulations. Each formulation represents a combination of DGEVA and a specific length (0.2 mm, 0.5 mm, or 2 mm) and amount (1, 2.5, 7, 15, or 25 phr) of RCFs. All the formulations were prepared using 1 phr of TPED and a 1:1 molar ratio of PIFA.

**Scheme 2:**
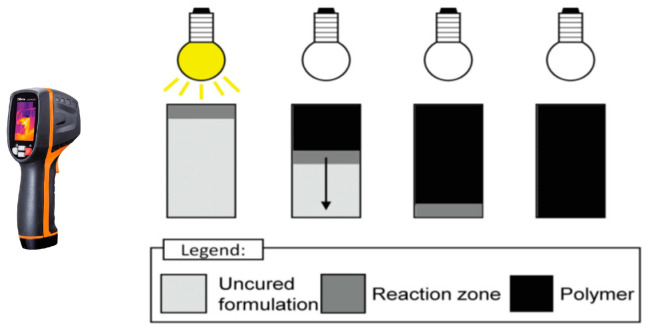
Scheme of the registration of the thermal front propagation towards a thermos-camera.

*Evaluation of frontal polymerization*: For the Radical-Induced Cationic Frontal Polymerization (RICFP) front assessment, a silicon mold was used for polymerization of dimensions: 10 mm × 10 mm × 60 mm. The reaction was initiated using a Hamamatsu LC8 lamp, which has a light intensity of 100 mW/cm^−2^. The characteristics of the front were examined using a FLIR E5 thermal camera, which has a thermal sensitivity of 0.1 °C. The thermal camera was set to record the temperature of specific spots, at different time intervals (see Scheme 2). The experiments were performed three times for each manufacturing condition.

*Dynamic Mechanical Thermal Analysis (DMTA)*: The thermomechanical characteristics of the thermosets were examined using a DMTA instrument from Triton Technology. The analysis was conducted over a 25 to 250 °C temperature range, at a heating rate of 3 °C/min. The instrument exerted a uniaxial oscillatory tensile stress at a frequency of 1 Hz, with a displacement set at 0.02. The analysis allows for the assessment of the viscoelastic properties of the material, yielding information on the storage and loss moduli (the elastic component—E′, and the viscous component—E″, respectively). The measurement was performed to estimate the glass transition (Tg), determined as the peak of the damping factor curve, tan *δ* (as E″/E′). The samples were UV-cured in a silicon mold with average dimensions of 1 × 5 × 25 mm. Measurements were conducted in triplicate.

*Photo Dynamic Scanning Calorimetry (photo-DSC)*: The photo-curing process was investigated using a Mettler TOLEDO DSC-1, outfitted with a Gas Controller GC100. The DSC was equipped with a mercury lamp, Hamamatsu LIGHTINGCURE LC8 (Hamamatsu Photonics), which utilized an optic fiber to directly irradiate the sample and the reference crucibles. The UV-light emission was centered at 365 nm, with an intensity of approximately 100 mW/cm^2^. Roughly 5–10 mg of the photocurable formulation was placed in an open aluminum pan (40 μL), while an empty pan served as the reference. The tests were conducted at 60 °C under a controlled atmosphere of N2 flow of 40 mL/min. The samples were irradiated twice for 10 min each, to adequately evaluate the UV-curing. The second run was performed to confirm complete curing and establish the baseline. The second curve was subtracted from the first to yield the curve associated exclusively with curing. The integration of this curve provided the heat release during the curing process. All data were analyzed using Mettler Toledo STARe software V9.2.

*Electrical conductivity*: The electrical conductivity tests were carried out according to ASTM D257 standard by using a Keithley 2410 source-meter. The dimensions of the specimens were 10 × 10 × 1 mm^3^. Silver conductive paint was used in two 10 × 1 mm^2^ opposite faces of the specimen to minimize the contact resistance. Here, the electrical resistance was calculated from the slope of the voltage-intensity (V-I) plot, sweeping the voltage from 0 to 3 V. Then, the electrical conductivity (σ) was calculated from Equation (1), where L is the distance between the electrodes and A the cross-sectional area.

σ = L/(A·R)(1)

*Joule heating*: The Joule heating characterization was carried out by using a FLIR T530 thermal camera while applying voltage ranging from 1 to 25 V using the same source-meter equipment as in the electrical characterization tests. Here, the maximum temperature, Tmax, and the average temperature, Tav, were recorded once the temperature was stable on the time for each applied voltage.

## 3. Results

Conductive, fully bio-based composites were synthesized through UV-activated Radical-Induced Cationic Polymerization (RICFP) utilizing a bio-based epoxy matrix derivatized from biomass, functionalized with epoxy groups, and strengthened with recycled carbon fibers. The initial step of the RICFP mechanism involves UV irradiation, which triggers the highly exothermic opening of epoxy rings. Subsequently, the heat released initiates frontal polymerization by inducing the dissociation of the radical thermal initiator. The resulting carbon-centered radicals undergo oxidation to form carbocations in the presence of the iodonium salt. These carbocations then propagate the cationic ring-opening polymerization process throughout the sample thickness until the front heat becomes unsustainable [34].

### 3.1. Photo Dynamic Scanning Calorimetry

**Figure 2:**
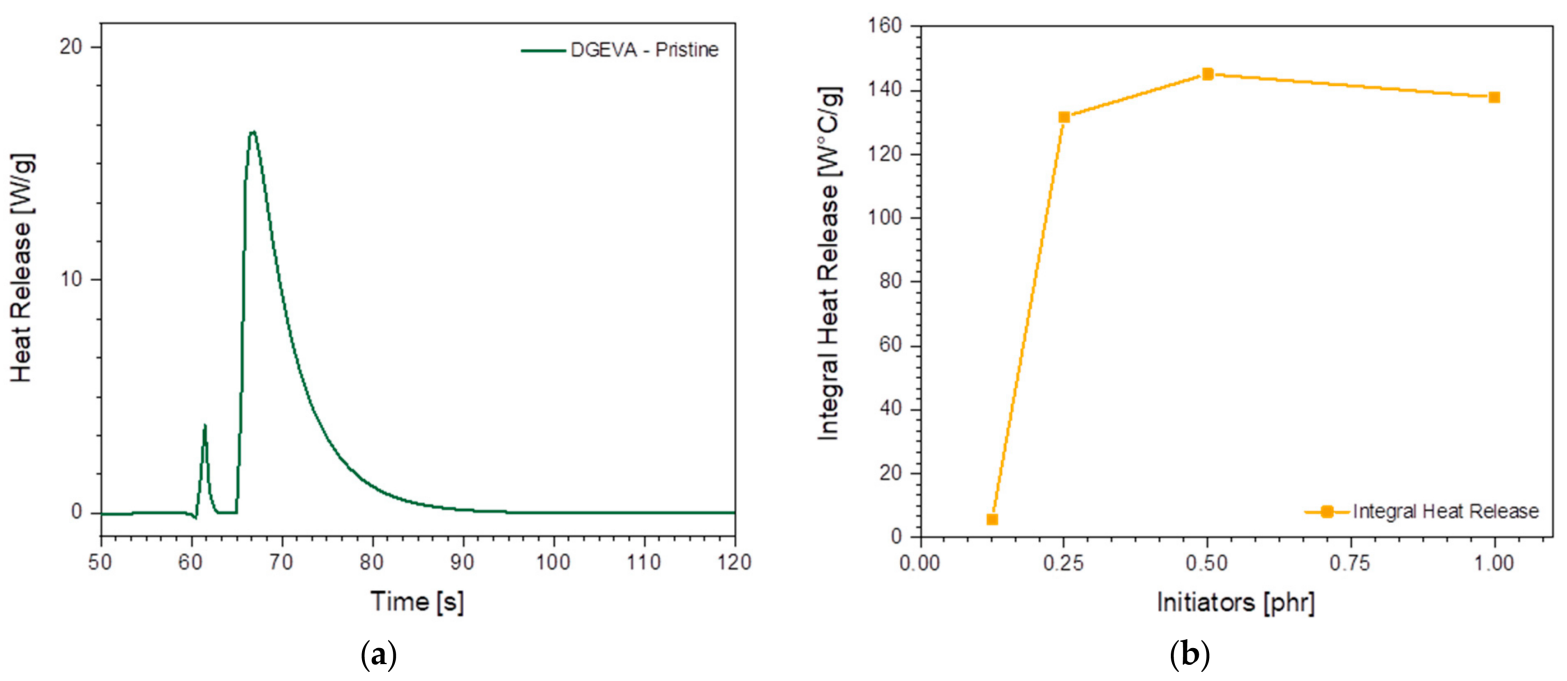
Photo-DSC measurements: heat release of pristine DGEVA formulation (**a**); variation of heat release (**b**).

Photo-DSC was performed in order to assess kinetics and the optimal amount of photo- and thermal-initiators. Figure 2a shows the heat release of the pristine DGEVA formulation with 1 phr of TPED and [TPED]/[PIFA] = 1.0 mol/mol. It can be observed that, as soon as the UV light is switched on, for a duration of 3 s, a small initiation peak appears, following the polymerization by frontal propagation. To maintain the thermal front, and to obtain optimal samples, the aforementioned initiators content was used.

As soon as the RCFs were added, the light penetration and absorption, combined with the heat dissipation, hindered the frontal polymerization, as reported in a previous work [35].

### 3.2. Thermal Font Propagation

**Figure 3:**
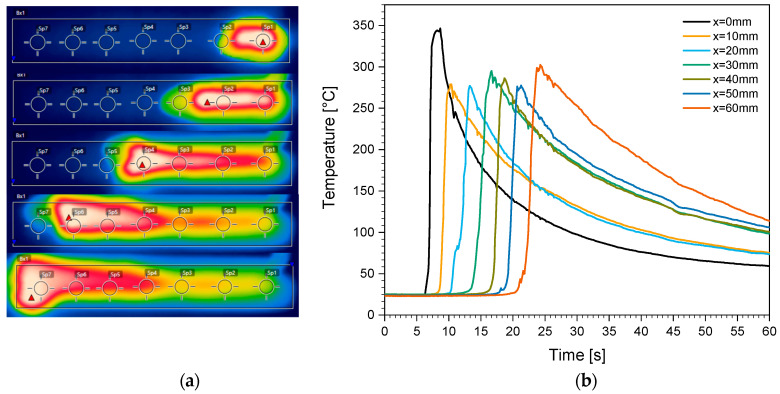
Thermal camera frame of frontal propagation (**a**); thermal camera measurements of pristine DGEVA formulation of temperature as function of time (**b**).

The frontal propagation of the bio-based epoxy formulations was assessed by a thermal camera, and the temperature was monitored at seven different points of the silicon mold. Pristine formulation was used to set the optimal amount and ratio between the photo-initiator and the thermal one. In total, 1 phr of TPED and [TPED]/[PIFA] = 1.0 mol/mol was used on the basis of the photo-DSC investigation and to prove the frontal propagation. In Figure 3, the thermal camera images are reported and the measured thermal front.

**Figure 4:**
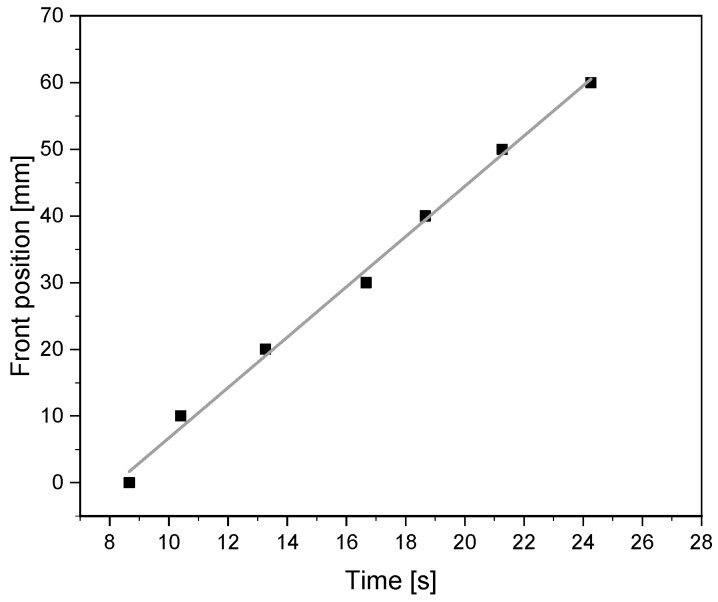
Front position as a function of time of the DGEVA pristine formulation.

The maintenance of the polymerization front was confirmed by the measurement of the front velocity at different points. Considering the front position as a function of time, as reported in Figure 4, it is evident that the front propagated at a constant rate.

Although the pristine formulation presented a front propagation mechanism, as soon as the RCFs were added to the formulation, the high thermal conductivity of the fibers dissipated the heat released, bringing the polymerization front to a halt. In order to sustain the front propagation, and to prevent the excess dissipation of the highly conductive filler, heating was assured to produce the 10 × 10 × 1 mm^3^ samples for the electrical conductivity tests, as also shown in previous works [35,36].

### 3.3. Dynamic Mechanical Thermal Analysis

**Figure 5:**
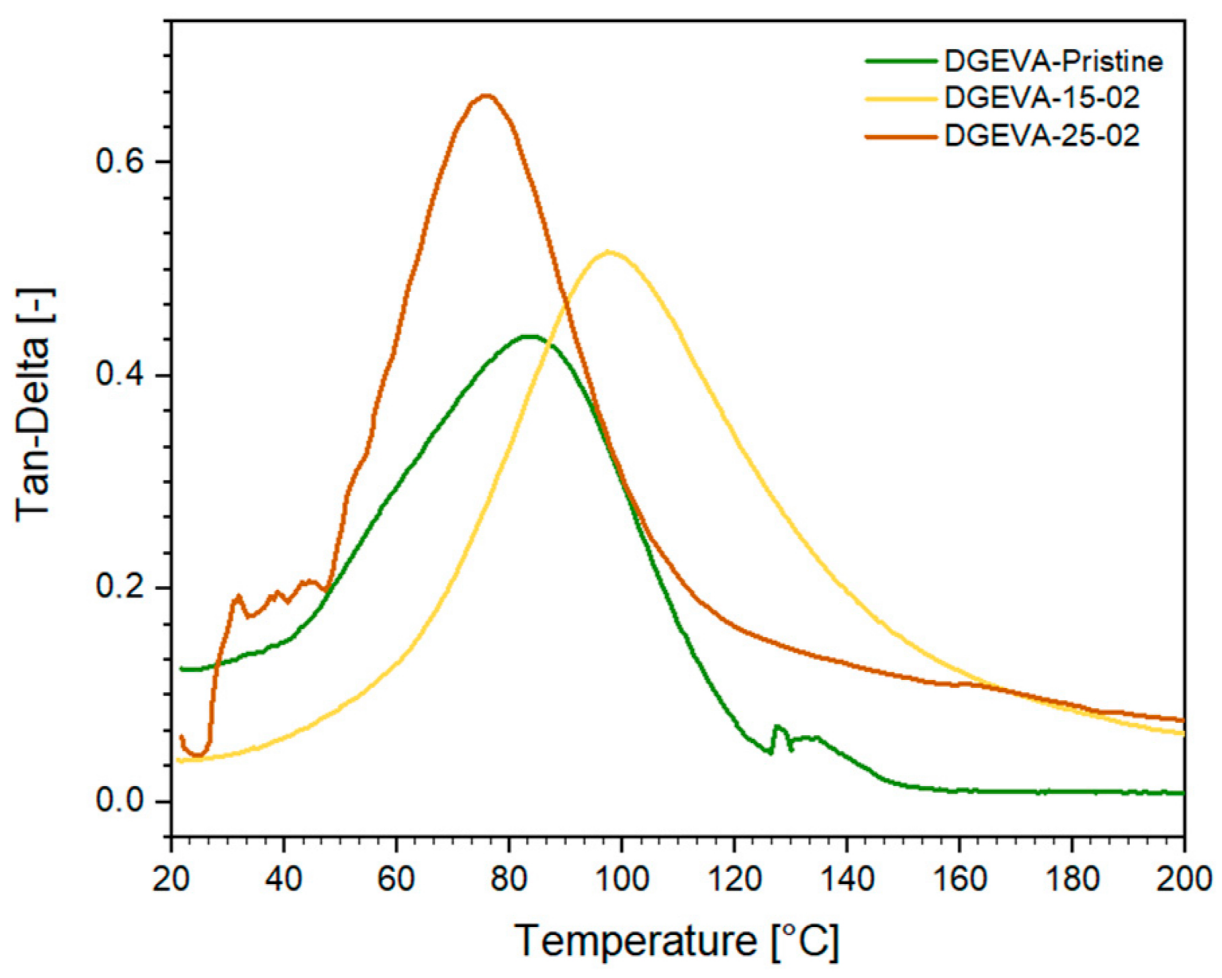
DMTA measurements of pristine formulation (green) and RCF-containing formulation.

The thermo-mechanical proprieties of the fully bio-based formulations were analyzed by means of DMTA on pristine samples and composites for different fiber lengths and concentrations. As an example, in Figure 5, the tanδ curves are reported for the frontal cured pristine DGEVA resin and for the DGEVA-based formulations containing, respectively, 15 phr and 25 phr of RCFs obtained using the 0.2 mm sieve.

The maximum of the tanδ peak is used to estimate the glass transition temperature Tg of the crosslinked samples. As can be observed in Figure 4, the Tg shifts towards a higher temperature as the RCFs are added to the pristine resin, going from 83 °C of the pristine formulation to a maximum of 97 °C with 15 phr of RCF. A further increase in the amount of RCFs showed a detrimental effect on Tg value, assessing to 75 °C with 25 phr of RCF. A similar trend was observed with different lengths of the fibers.

### 3.4. Electrical Conductivity

**Figure 6:**
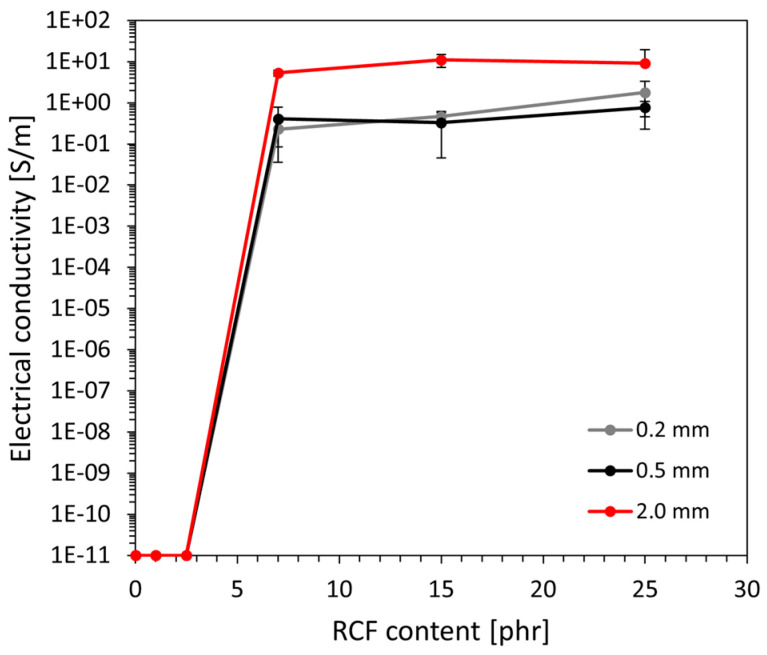
Electrical conductivity as a function of the RCF content and sieve used in the milling process, dispersed into the DGEVA matrix and frontal cured.

The DGEVA-based frontal-cured composites were characterized in terms of their electrical conductivity as a function of RCF content and length. The results of the electrical conductivity tests are shown in Figure 6. Here, the electrical percolation threshold is found to be between 2.5 phr and 7.0 phr, regardless of the sieve used in the milling process. Furthermore, the composites containing RCFs obtained with the 2.0 mm sieve showed a higher electrical conductivity than the ones obtained with the smaller sieves, 0.5 and 0.2 mm, which showed a similar behavior. This can be explained due to the electrically conductive network created by the RCFs. In this context, the electrical conductivity is dominated by the intrinsic resistance of the carbon fibers upon the electrical percolation threshold, rather than the contact and tunneling mechanisms [37]. Therefore, the conductive network created with the longest fibers presents a higher continuity and, thus, a lower electrical resistance.

### 3.5. Joule Heating Effect

**Figure 7:**
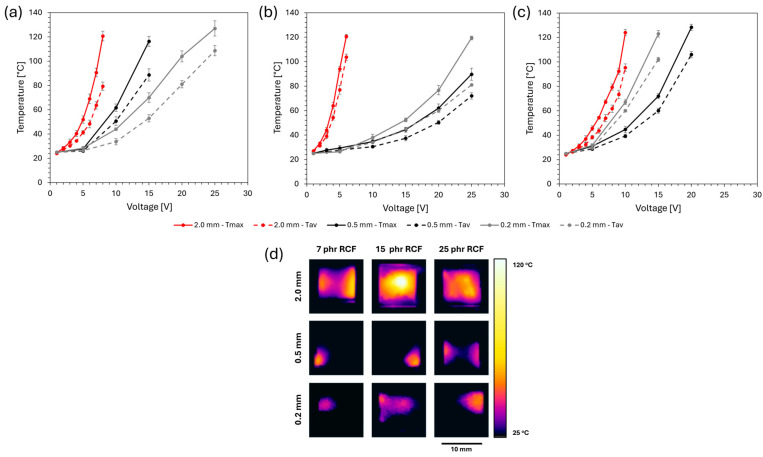
Maximum and average temperatures reached by Joule effect as a function of the applied voltage for the specimens containing (**a**) 7 wt.%RCF, (**b**) 15 wt.%RCF, and (**c**) 25 wt.%RCF. (**d**) Thermographs taken at 6 V for all tested conditions.

As expected, the Joule heating behavior of the developed composites is directly related to the electrical conductivity (see Figure 7). More specifically, Figure 7a–c shows the Tmax and Tav reached by Joule heating as a function of the applied voltage for the specimens containing a 7 phr RCF, 15 phr RCF, and 25 phr RCF, respectively. In this regard, a higher electrical conductivity results in a higher current intensity for the same applied voltage. Thus, considering the Joule law (see Equation (2)), the heat released by Joule effect, Q, is directly proportional to the voltage, the intensity, and the time (t).

Q = I · V · t(2)

In this context, the composites containing the longest fibers, obtained with the 2.0 mm sieve, showed the highest temperatures for the same applied voltage, being able to reach maximum temperatures of around 120 °C by applying just 6 to 10 V. On the other hand, the composites containing the RFC obtained with the smaller sieves showed poorer Joule heating capabilities due to the lower electrical conductivity. Here, the voltage needed to reach a maximum temperature of around 120 °C ranges from 15 to 30 V.

Figure 7d shows the thermographs of the developed composites for qualitative comparison purposes. All of the thermographs were taken using 6 V, since it was the maximum common voltage for all tested conditions. In this sense, the higher temperatures reached by the composites containing the 2.0 mm sieve RCF are evinced. Moreover, considering the temperature distribution of the composite containing the 2.0 mm sieve RCF, the ones containing 15 phr RCF showed a higher homogeneity. This can be explained, since the ones with a lower RCF content, 7 phr RCF, are close to the electrical percolation threshold, presenting some preferred conductive pathways. In contrast, the ones with a higher RCF content, 25 phr RCF, are close to the manufacturability limit, due to the high viscosity of the mixture, hindering the dispersion process and, thus, leading to RCF aggregation.

## 4. Conclusions

Bio-based sustainable conductive epoxy composites were achieved by exploiting the RICFP process. The bio-based DGEVA precursor was used as a matrix, and mechanically recycled carbon fibers were dispersed in order to achieve electrically conductive composites. Both materials and process can be considered sustainable, since a bio-based epoxy precursor was derived from agro-food waste and epoxy functionalized. The conductive carbon fibers were mechanically recycled from expired off-cuts generated during the manufacturing process of continuous fiber-reinforced polymers (CFRP) from carbon fiber-epoxy prepregs. The UV-induced frontal polymerization process was proposed as a sustainable method for composite production, due to its low energy consumption.

Photo-DSC was used to define the optimal amount of photo- and thermal-initiators to promote the UV-induced frontal polymerization process. The formulation with 1 phr of TPED and [TPED]/[PIFA] = 1.0 mol/mol ratio was used in the following investigations. The frontal propagation of the bio-based epoxy formulations was assessed by a thermal camera, and the temperature was monitored at seven different points. The maintenance of the polymerization front was confirmed for the pristine resin, while heating of the sample during irradiation was required for the composite formulations, due to their higher thermal conductivity, leading to a faster heat dissipation. Nevertheless. the frontal process was assured for all the investigated formulations containing RCFs.

The frontal-cured composites were characterized by DMTA, showing an enhancement of the Tg value up to a content of 15 phr fibers, and a following decrease by further increasing the fiber content.

The electrical conductivity tests showed a percolation threshold for the cured composites between 2.5 phr and 7.0 phr, regardless of the sieve used in the milling process. The composites containing RCFs obtained with the 2.0 mm sieve showed a higher electrical conductivity than the ones obtained with the smaller sieves, 0.5 and 0.2 mm, due to their higher aspect ratio. As a consequence, a different Joule heating behavior was measured. In fact, the Joule heating behavior of the developed composites is directly related to their electrical conductivity. In this context, the composites containing the longest fibers, obtained with the 2.0 mm sieve, showed the highest temperatures for the same applied voltage, being able to reach maximum temperatures of around 120 °C by applying just 6 to 10 V. On the other hand, the composites containing the RFC obtained with the smaller sieves showed poorer Joule heating capabilities, due to the lower electrical conductivity. Here, the voltage needed to reach a maximum temperature of around 120 °C ranges from 15 to 30 V.

In conclusion, in this paper we have prepared sustainable conductive bio-based epoxy composites following circular economy production, exploiting derivatized bio-based epoxy precursors and mechanically recycled carbon fibers. The conductive composites showed interesting Joule heating capabilities, which can be further used in different applications, such as antifog or anti-icing and de-icing systems, thermotherapy devices, or for triggering a shape memory cycle, among others.

## Figures and Tables

**Table 1:** Prepared formulations.

RCF Amount	0.2 mm (RCF Lenght)	0.5 mm (RCF Lenght)	2 mm (RCF Lenght)
1 phr	DGEVA-0.2 mm-1 phr	DGEVA-0.5 mm-1 phr	DGEVA-2 mm-1 phr
2.5 phr	DGEVA-0.2 mm-2.5 phr	DGEVA-0.5 mm-2.5 phr	DGEVA-2 mm-2.5 phr
7 phr	DGEVA-0.2 mm-7 phr	DGEVA-0.5 mm-7 phr	DGEVA-2 mm-7 phr
15 phr	DGEVA-0.2 mm-15 phr	DGEVA-0.5 mm-15 phr	DGEVA-2 mm-15 phr
25 phr	DGEVA-0.2 mm-25 phr	DGEVA-0.5 mm-25 phr	DGEVA-2 mm-25 phr

## Data Availability

The original contributions presented in the study are included in the article, further inquiries can be directed to the corresponding author/s.
